# Supplementary material for: Postnatal development of the dentate gyrus vascular niche
Source: Sci Rep. 2025 Nov 4;15:38550. doi: 10.1038/s41598-025-22591-1 (PMC12586690; doi:10.1038/s41598-025-22591-1)
Supplement: Supplementary file 1 — Supplementary Material 1 [file 41598_2025_22591_MOESM1_ESM.pdf]

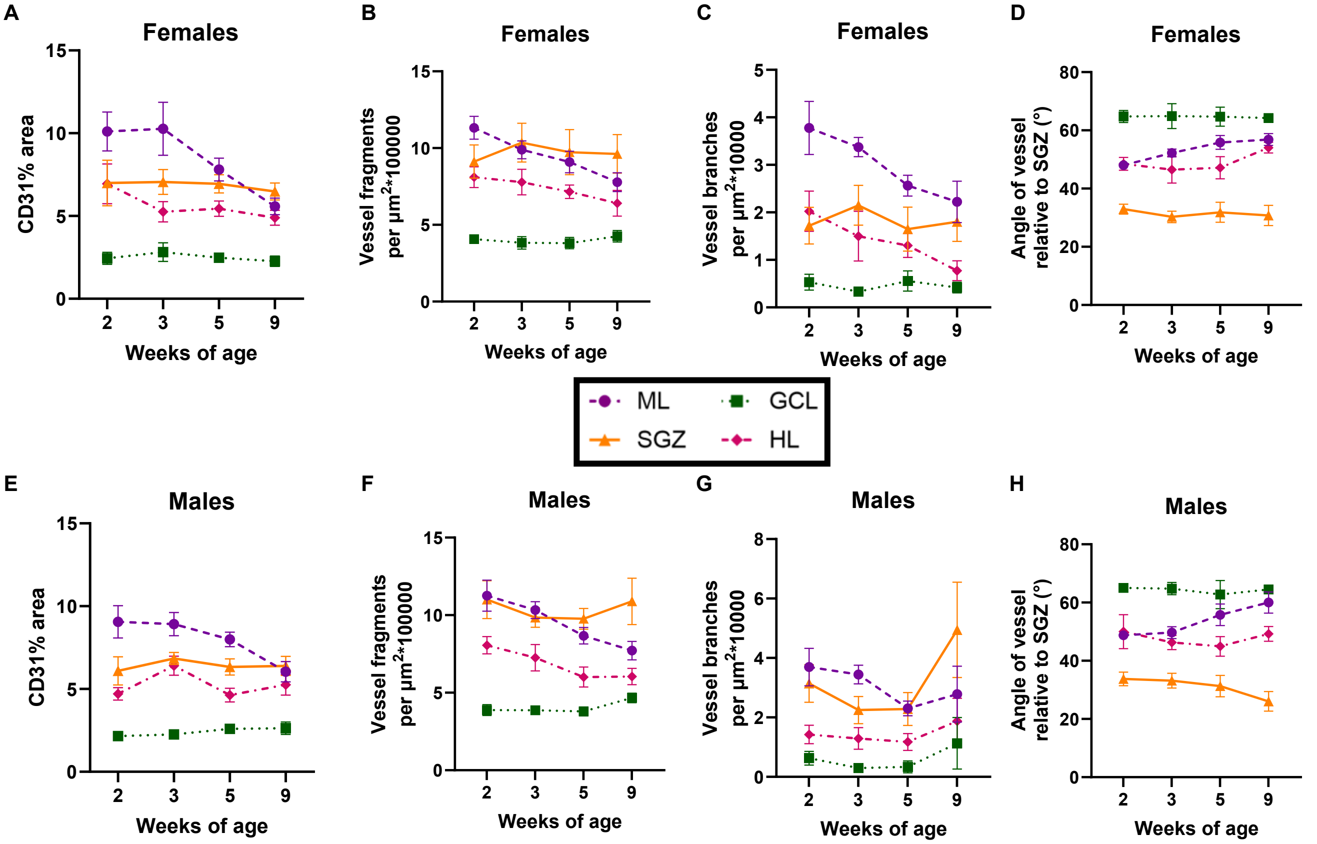

Fig S1: Vascular architecture in the postnatal DG by sex. **(A), (E)** Proportion of DG area covered by CD31 immunolabeling. 3-way repeated measures ANOVA age x layer x sex ($F_{9,108}$)=2.56, p=0.02; age x layer ($F_{9,108}$)=9.95, p<0.001; layer x sex ($F_{3,108}$)=0.39, p=0.76; age x sex ($F_{3,36}$)=0.59, p=0.69; age ($F_{3,36}$)=2.08, p=0.12; sex ($F_{1,36}$)=0.71, p=0.40; layer ($F_{3,108}$)=332.85, p<0.001. **(B), (F)** Density of vessel fragments. 3-way repeated measures ANOVA age x layer x sex ($F_{6.94,83.24}$)=0.39, p=0.91; age x layer ($F_{6.94,83.24}$)=2.91, p=0.009; layer x sex ($F_{2.31,83.24}$)=1.07, p=0.36; age x sex ($F_{3,36}$)=0.24, p=0.87; age ($F_{3,36}$)=2.1, p=0.12; sex ($F_{1,36}$)=0.01, p=0.91; layer ($F_{2.31,83.24}$)=136.26, p<0.001. **(C), (G)** Density of branch points. 3-way repeated measures ANOVA age x layer x sex ($F_{6.52,78.21}$)=0.89, p=0.51; age x layer ($F_{6.52,78.21}$)=2.64, p=0.019; layer x sex ($F_{2.17,78.21}$)=4.86, p=0.009; age x sex ($F_{3,36}$)=1.61, p=0.20; age ($F_{3,36}$)=0.92, p=0.44; sex ($F_{1,36}$)=2.34, p=0.14; layer ($F_{2.17,78.21}$)=63.62, p<0.001. **(D), (H)** Orientation of vessels relative to SGZ. 3-way repeated measures ANOVA age x layer x sex ($F_{9,108}$)=0.44, p=0.91; age x layer ($F_{9,108}$)=2.99, p=0.003; layer x sex ($F_{3,108}$)=0.27, p=0.84; age x sex ($F_{3,36}$)=0.67, p=0.58; age ($F_{3,36}$)=1.81, p=0.16; sex ($F_{1,36}$)=1.73, p=1.20; layer ($F_{3,108}$)=165.91, p<0.001. **(A-H)** Mean ± SEM shown throughout with n = individual mice. 2 weeks (n=10, 5M/5F), 3 weeks (n=12, 8M/4F), 5 weeks (n=11, 5M/6F) and 9 weeks (n=11, 5M/6F).

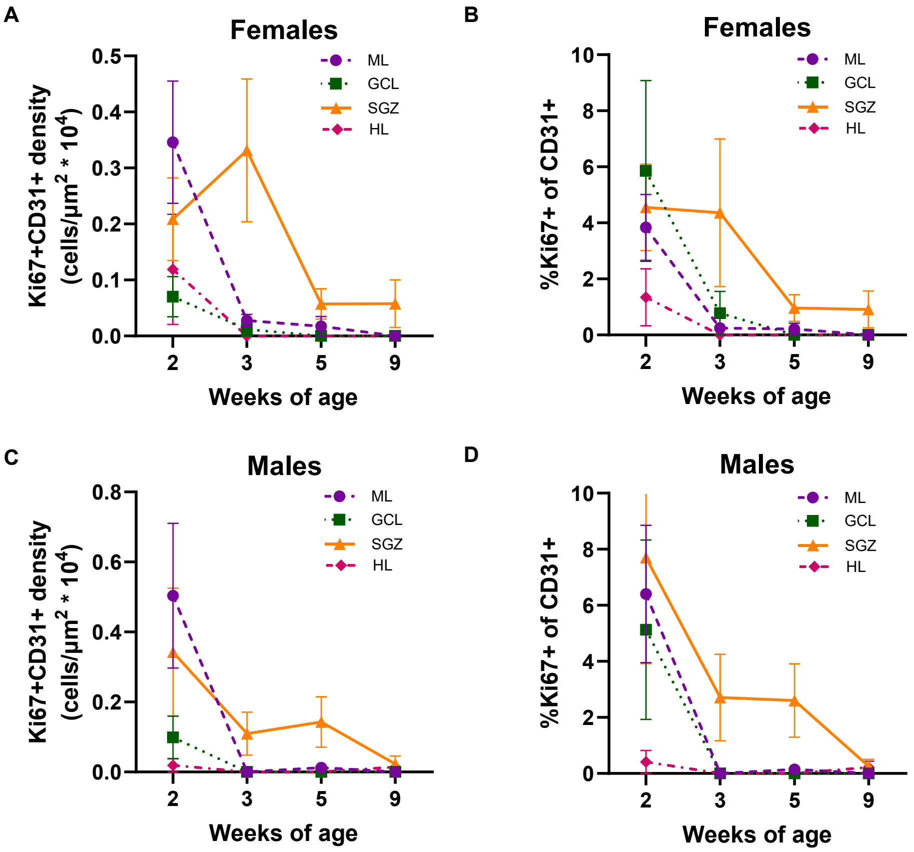

Fig S2: Angiogenesis levels in the postnatal DG by sex. **(A), (C)** Density of CD31+/Ki67+ double labeled cells. 3-way repeated measures ANOVA age x layer x sex ($F_{6.18,74.16}$)=1.47, p=0.2; age x layer ($F_{6.18,74.16}$)=5.46, p<0.001; layer x sex ($F_{2.06,74.16}$)=0.36, p=0.70; age x sex ($F_{3,36}$)=0.82, p=0.49; age ($F_{3,36}$)=10.59, p<0.001; sex ($F_{1,36}$)=0.002, p=0.97; layer ($F_{2.06,74.16}$)=13.68, p<0.001. **(B), (D)** Proportion of CD31+ cells co-labeled with Ki67. 3-way repeated measures ANOVA age x layer x sex ($F_{5.41,64.92}$)=0.64, p=0.68; age x layer ($F_{5.41,64.92}$)=2.20, p=0.06; layer x sex ($F_{1.80,64.92}$)=0.41, p=0.65; age x sex ($F_{3,36}$)=0.40, p=0.75; age ($F_{3,36}$)=11.55, p<0.001; sex ($F_{1,36}$)=0.07, p=0.79; layer ($F_{1.80,64.92}$)=8.25, p<0.001. **(A-D)** Mean ± SEM shown throughout with n = individual mice. 2 weeks (n=10, 5M/5F), 3 weeks (n=12, 8M/4F), 5 weeks (n=11, 5M/6F) and 9 weeks (n=11, 5M/6F).a

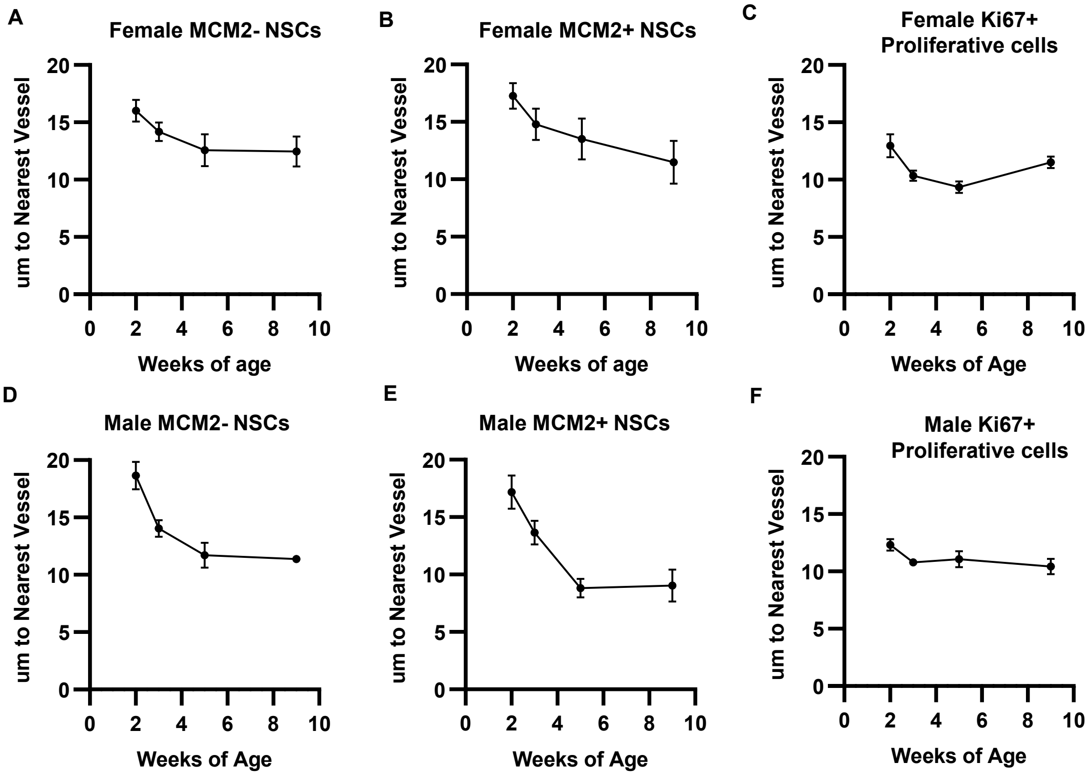

Fig S3. Average distance of MCM2- NSCs, MCM2+ NSCs, and Ki67+ proliferative cells to nearest CD31+ vessel by sex. (**A**), (**D**) MCM2- NSCs average distance to nearest CD31+ vessel. 2-way repeated measures ANOVA (age x sex), Age x sex $F_{(3,34)} = 1.278$, $p=0.2975$; age $F_{(3,34)} = 10.76$, $p<0.0001$; sex $F_{(1,34)} = 0.005830$, $p=0.9396$. (**B**), (**E**) MCM2+ NSCs average distance to nearest CD31+ vessel. 2-way repeated measures ANOVA (age x sex), Age x sex $F_{(3,34)} = 0.9905$, $p=0.4089$; age $F_{(3,34)} = 8.974$, $p=0.0002$; sex $F_{(1,34)} = 3.516$, $p=0.0694$. (**C**), (**F**) Ki67+ Proliferative cells average distance to nearest CD31+ vessel. 2-way repeated measures ANOVA (age x sex), Age x sex $F_{(3,34)} = 2.149$, $p=0.1122$; age $F_{(3,34)} = 6.412$, $p=0.0015$; sex $F_{(1,34)} = 0.07872$, $p=0.7807$. **(A-E)** Mean ± SEM shown throughout with n = individual mice. 2 weeks (n=10, 5M/5F), 3 weeks (n=12, 8M/4F), 5 weeks (n=11, 5M/6F) and 9 weeks (n=11, 5M/6F).

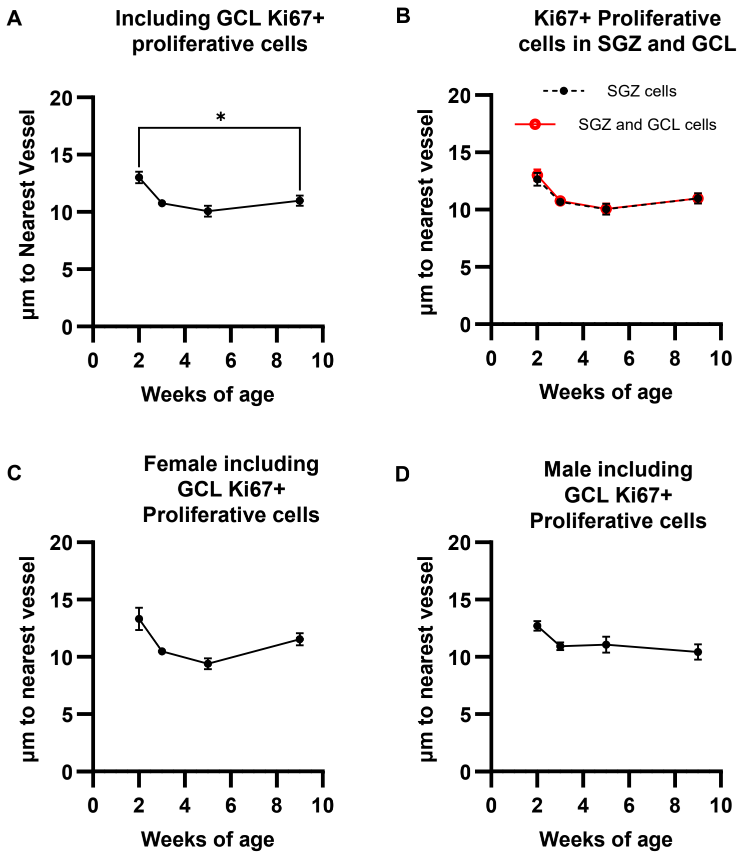

Fig S4. Average distance of Ki67+ proliferative cells located in the GCL and SGZ to nearest CD31+ vessel. (**A**) Ki67+ proliferative cells located in the SGZ and GCL average distance to nearest CD31+ vessel. One-way ANOVA p=0.0001, F(3,38) = 8.88, **-p=0.005 from Dunnett's multiple comparisons to 9 weeks of age. (**B**) Comparing Ki67+ proliferative cells located only in the SGZ and in the SGZ and GCL average distance to nearest CD31+ vessel **(C**), (**D**) Ki67+ proliferative cells located in the SGZ and GCL average distance to nearest CD31+ vessel separated by sex. 2-way ANOVA (age x sex), Age x sex $F_{(3,34)}$ = 2.18, p=0.11; age $F_{(3,34)}$ = 8.80, p=0.0002; sex $F_{(1,34)}$ = 0.05, p=0.83. **(A-D)** Mean ± SEM shown throughout with n = individual mice. 2 weeks (n=10, 5M/5F), 3 weeks (n=12, 8M/4F), 5 weeks (n=11, 5M/6F) and 9 weeks (n=11, 5M/6F).

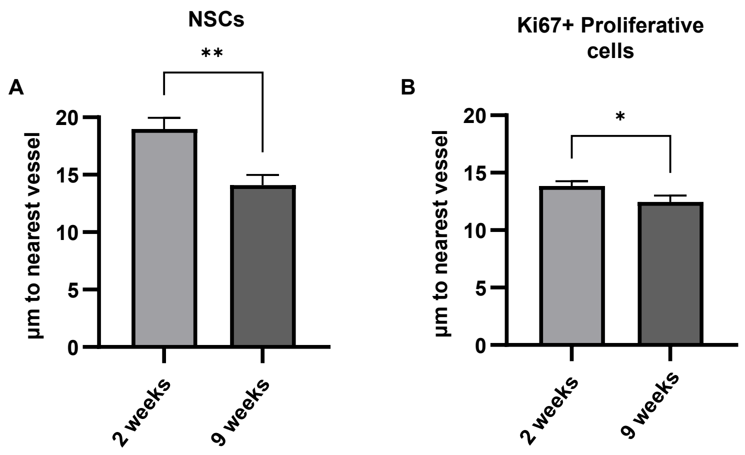

Fig S5. Average 3-Dimensional (3D) distance of NSCs and Ki67+ Proliferative cells to nearest blood vessel at 2 and 9 weeks of age (**A**) NSCs average 3D distance to nearest blood vessel. Mann-Whitney U test, **- p=0.0015, U=10. **(B)** Ki67+ proliferative cells average 3D distance to nearest blood vessel. Mann-Whitney U test, *- p=0.0433, U=23. **(A,B)** (n= mice) 2 weeks (n=10, 5M/5F) and 9 (n=10, 5M/5F) weeks.

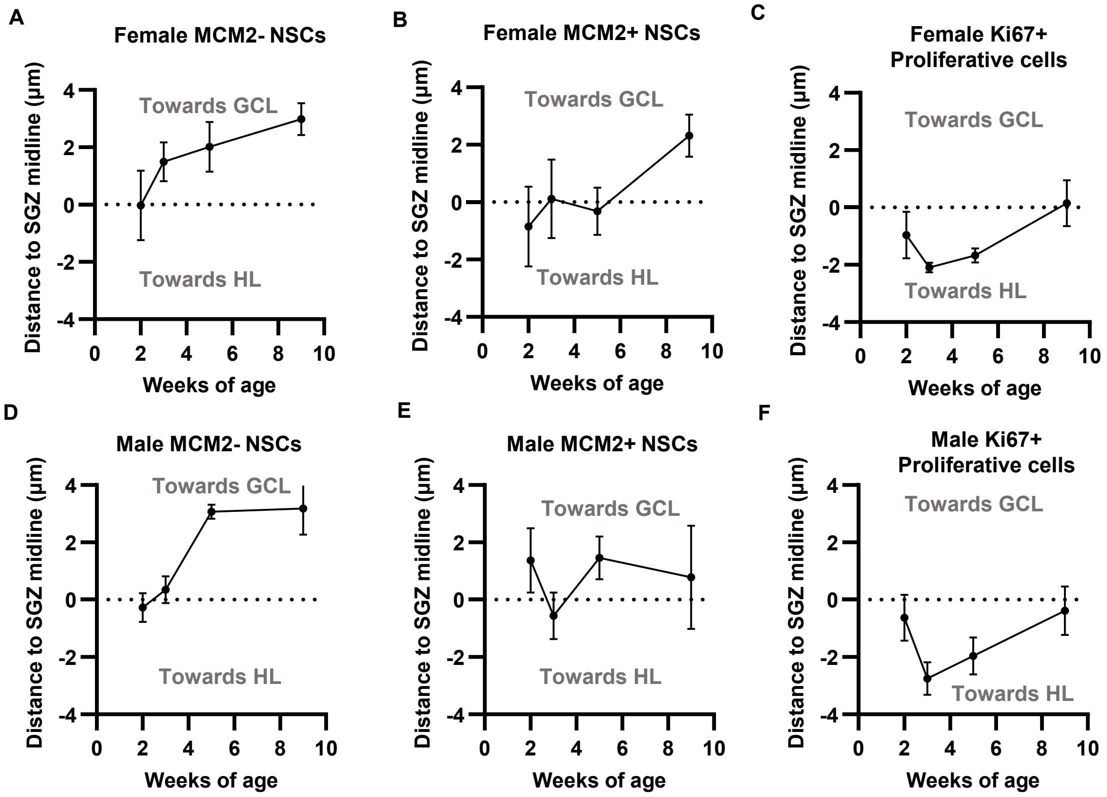

Fig S6. Average distance of MCM2- NSCs, MCM2+ NSCs, and Ki67+ Proliferative cells to SGZ midline by sex (**A**), (**D)** MCM2- NSCs average distance to nearest SGZ midline. 2-way repeated measures ANOVA (age x sex), Age x sex $F_{(3,34)}$ = 0.4429, p=0.7239; age $F_{(3,34)}$ = 7.730, p=0.0005; sex $F_{(1,34)}$ = 0.02913, p=0.8655. (**B**), (**E**) MCM2+ NSCs average distance to nearest SGZ midline. 2-way repeated measures ANOVA (age x sex), Age x sex $F_{(3,34)}$ = 1.343, p=0.2768; age $F_{(3,34)}$ = 0.8839, p=0.4592; sex $F_{(1,34)}$ = 0.2393, p=0.6278. **(C),** (**F**) Ki67+ Proliferative cells average distance to nearest SGZ midline. 2-way repeated measures ANOVA (age x sex), Age x sex $F_{(3,33)}$ = 0.3217, p=0.8096; age $F_{(3,33)}$ = 4.612, p=0.0084; sex $F_{(1,33)}$ = 0.5673, p=0.4567**. (A-F)** (n= mice) 2 (n=10, 5M/5F), 3 (n=12, 8M/4F), 5 (n=10, 4M/6F) and 9 (n=10, 5M/5F) weeks.

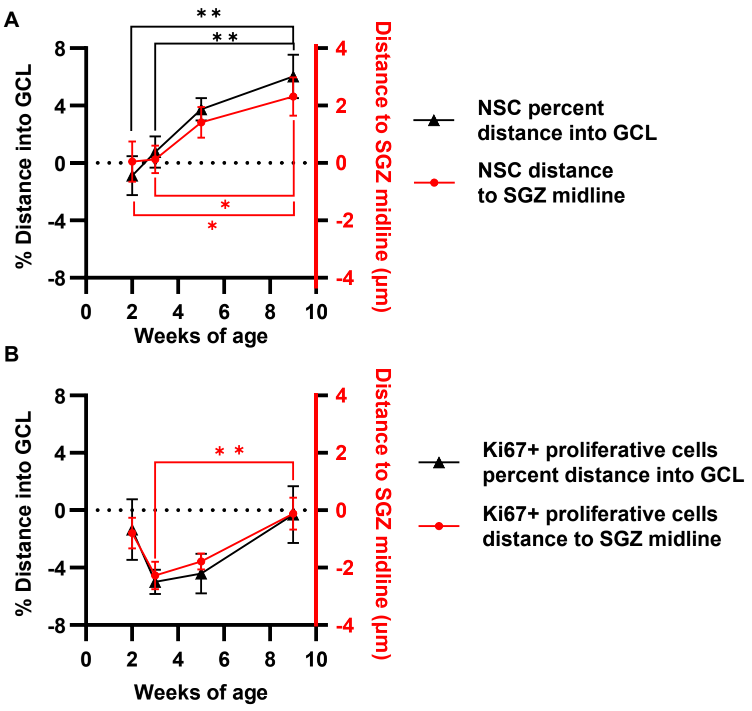

Fig S7. Average distance of NSCs and Ki67+ Proliferative cells to SGZ midline compared to average percent distance into GCL of NSCs and Ki67+ Proliferative cells (**A**) NSCs average percent distance into GCL relative to total GCL width is plotted on the left y axis. The raw distance to the SGZ midline from Fig 3 is plotted on the right axis for comparison. Percent distance: one-way ANOVA p=0.0015, $F_{(3,38)}$ =6.212. 9 vs 2 weeks of age **- p=0.0010, 9 vs 3 weeks of age **- p=0.0095 from Dunnett's multiple comparisons to 9 weeks of age. Raw distance to SGZ: one-way ANOVA p=0.0293, $F_{(3,38)}$ =3.337. 9 vs 2 weeks of age *- p=0.0324, 9 vs 3 weeks of age *- p=0.0300 from Dunnett's multiple comparisons to 9 weeks of age. (**B**) Ki67+ proliferative cells average percent distance into GCL relative to total GCL width is plotted on the left y axis. The raw distance to the SGZ midline from Fig 3 is plotted on the right axis for comparison. Percent distance: One-way ANOVA p=0.1228, $F_{(3,38)}$ =2.052. Raw distance to SGZ: One-way ANOVA p=0.0121, $F_{(3,38)}$ =4.158. **- p=0.0069 from Dunnett's multiple comparisons to 9 weeks of age. **(A,B)** (n= mice) 2 (n=10, 5M/5F), 3 (n=12, 8M/4F), 5 (n=10, 4M/6F) and 9 (n=10, 5M/5F) weeks.
